# Diverticular per oral endoscopic septotomy with selective crurotomy as salvage therapy for severe esophagogastric junction outflow obstruction after endoscopic and surgical therapy

**DOI:** 10.1055/a-2852-7295

**Published:** 2026-05-05

**Authors:** Laith Eyad Baqain, Kaleb Bogale, Nabil Noureddin, Philip Katz, David Carr-Locke, Reem Z. Sharaiha, Kartik Sampath

**Affiliations:** 1 Division of Gastroenterology and HepatologyDepartment of Medicine 12295 NewYork-Presbyterian Weill Cornell Medical CenterNew YorkUnited States

Esophagogastric junction outflow obstruction (EGJOO) following prior cardiomyotomy and fundoplication represents a challenging clinical scenario, particularly when associated with an epiphrenic diverticulum. Surgical revision in this setting is technically demanding due to distorted anatomy, fibrosis, and the loss of standard tissue planes 1 . Diverticular peroral endoscopic myotomy (D-POEM) offers a minimally invasive alternative by addressing both the diverticular septum and the hypercontractile distal esophageal segment 2 . In select cases with prior myotomy, residual outflow obstruction may be attributable to extrinsic compression from the diaphragmatic crura, which can be addressed via a selective endoscopic crurotomy.

**Fig. 1:**
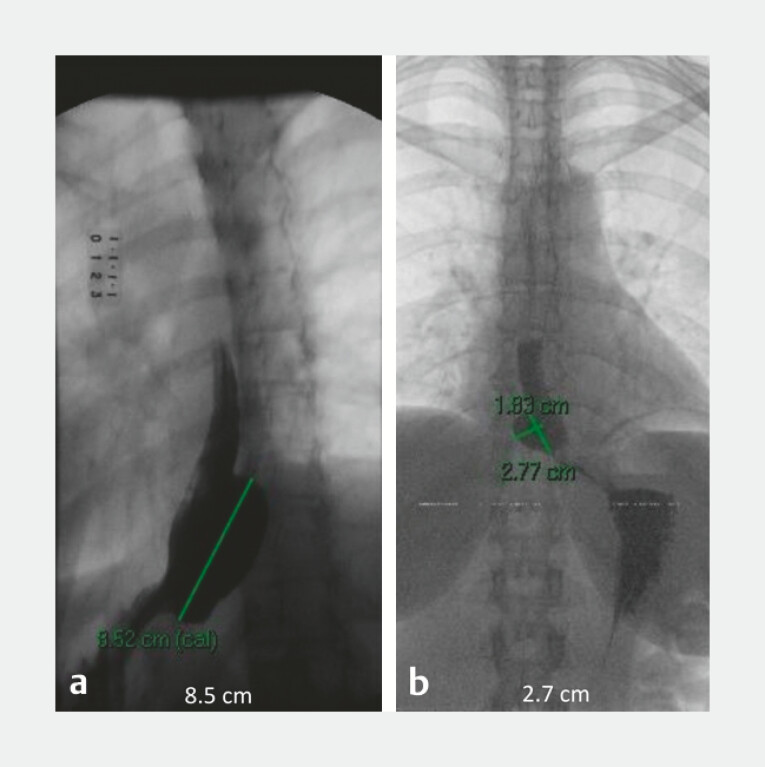
**a** Pre-procedural barium swallow demonstrated an 8.5cm epiphrenic diverticulum. **b** A post-procedural esophagram showing the reduction of the diverticulum to 2.7 cm.

A 48-year-old woman with lupus and hypercontractile esophagus previously underwent posterior POEM at an outside institution, complicated by severe gastroesophageal reflux disease and hiatal hernia requiring surgical repair with Toupet fundoplication. She subsequently developed progressive solid food dysphagia and an enlarging epiphrenic diverticulum despite surgical revision with partial loosening of the wrap. A timed barium esophagram demonstrated an 8.5-cm epiphrenic diverticulum, and EndoFLIP showed moderate to severely reduced gastroesophageal junction (GEJ) distensibility consistent with clinically significant EGJOO ( Fig. 1 **a** ).

**Fig. 2:**
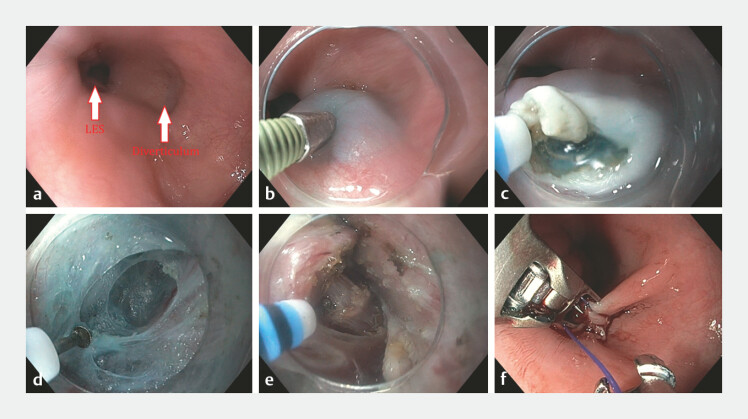
**a** An endoscopic view of a diverticulum proximal to the lower esophageal sphincter. **b** Submucosal injection with methylene blue. **c** Transverse mucosotomy **. d** A submucosal tunnel extended to 42cm through careful dissection. **e** Full thickness myotomy extended into gastric cardia. **f** Closure of the mucosal entry site using a single endoscopic suture.

Following multidisciplinary discussion, endoscopic therapy via D-POEM was pursued. Submucosal injection with saline and methylene blue was performed proximal to the diverticular septum ( Fig. 2 **a** and **b** ), followed by transverse mucosotomy ( Fig. 2 **c** ) and creation of a submucosal tunnel extending beyond the GEJ ( Fig. 2 **d** ). A full-thickness septotomy with cardiomyotomy was performed, with extension into the gastric cardia ( Fig. 2 **e** ). Given the minimal residual intrinsic muscle from the prior POEM and persistence of a prominent crural ledge, a selective endoscopic diaphragmatic crurotomy was performed to relieve extrinsic obstruction. Post-intervention EndoFLIP demonstrated marked improvement in GEJ distensibility. The mucosal entry was closed using endoscopic suturing ( Fig. 2 **f** , Video 1 ).

A post-procedural esophagram confirmed prompt contrast passage, the absence of leak, and immediate reduction in a diverticular size to 2.7 cm ( Fig. 1 **b** ). The patient recovered without adverse events and reported significant improvement in dysphagia. This case demonstrates the feasibility of combining D-POEM with selective endoscopic crurotomy as a minimally invasive salvage therapy for the management of a symptomatic epiphrenic diverticulum/EGJOO post-surgical hiatal hernia repair with fundoplication.

Diverticular peroral endoscopic septotomy with diaphragmatic crurotomy after prior endoscopic myotomy and surgical hiatal hernia repair with fundoplication, demonstrating tunneling, septotomy, cardiomyotomy, crurotomy, closure, and improved distensibility.Video 1

Endoscopy_UCTN_Code_TTT_1AO_2AP
